# Quantification of High-Molecular Weight Protein Platforms by AQUA Mass Spectrometry as Exemplified for the CD95 Death-Inducing Signaling Complex (DISC)

**DOI:** 10.3390/cells2030476

**Published:** 2013-06-27

**Authors:** Uwe Warnken, Kolja Schleich, Martina Schnölzer, Inna Lavrik

**Affiliations:** 1 Functional Proteome Analysis, German Cancer Research Center (DKFZ), 69120 Heidelberg, Germany; 2 Division of Immunogenetics, German Cancer Research Center (DKFZ), 69120 Heidelberg, Germany; 3 Department of Translational Inflammation Research, Institute of Experimental Internal Medicine, Otto von Guericke University, 39120 Magdeburg, Germany

**Keywords:** CD95, DISC, apoptosis, DED, protein quantification, mass spectrometry, AQUA technique, protein platforms

## Abstract

Contemporary quantitative mass spectrometry provides fascinating opportunities in defining the stoichiometry of high-molecular weight complexes or multiprotein platforms. The composition stoichiometry of multiprotein platforms is a key to understand the regulation of complex signaling pathways and provides a basis for constructing models in systems biology. Here we present an improved AQUA technique workflow that we adapted for the quantitative mass spectrometry analysis of the stoichiometry of the CD95 (Fas/APO-1) death inducing signaling complex (DISC). The DISC is a high-molecular weight platform essential for the initiation of CD95-mediated apoptotic and non-apoptotic responses. For protein quantification, CD95 DISCs were immunoprecipitated and proteins in the immunoprecipitations were separated by one-dimensional gel electrophoresis, followed by protein quantification using the AQUA technique. We will discuss in detail AQUA analysis of the CD95 DISC focusing on the key issues of this methodology, *i.e.*, selection and validation of AQUA peptides. The application of this powerful method allowed getting new insights into mechanisms of procaspase-8 activation at the DISC and apoptosis initiation [1]. Here we discuss the AQUA methodology adapted by us for the analysis of the CD95 DISC in more detail. This approach paves the way for the successful quantification of multiprotein complexes and thereby delineating the intrinsic details of molecular interactions.

## 1. Introduction

Most comparative proteomics studies deliver relative quantification data describing the changes in protein expression relative to another cellular state. However, in systems biology there is an increasing need for information about absolute numbers of expression levels of proteins including their posttranslational modifications [2,3]. Recently, new technologies such as AQUA [4] have been described that allow absolute quantification of proteins by mass spectrometry-based simultaneous determination of representative proteolytic peptides derived from the proteins of interest and their isotope-labeled analogs.

AQUA stands for “absolute quantification” and makes use of synthetic peptides with incorporated stable isotopes (^13^C, ^15^N) as internal standards. The sequences of these so-called “heavy” peptides are derived from the sequence of the proteins that are selected for quantification. The heavy peptides are spiked in at known amounts to protein mixtures of unknown concentrations. Upon proteolytic digestion of the proteins, the corresponding native or “light” peptides having the same sequence as the heavy AQUA peptides are generated. Since the heavy labeled AQUA peptide is chemically indistinguishable from its native light counterpart but differs in its molecular weight, mass spectrometry can be used as read-out to determine the ratio of heavy and light peptides (H/L ratio). The exact amount of spiked-in heavy peptide is known, thus, the amount of light peptide and thereby of the protein can be calculated from this H/L ratio. 

For determining the stoichiometry of already known interaction partners in protein complexes, a combination of complex isolation and quantitative proteomics using the AQUA strategy is a straightforward analytical approach [5].

Major decisions in signaling pathways are regulated by the formation of high molecular weight complexes or multiprotein platforms. Quantitative mass spectrometry has been successfully applied for the analysis of a number of complexes and signaling pathways, however, so far this application was limited [6,7]. In particular, protein platforms formed in the course of cell death have not been systematically analyzed.

**Figure 1:**
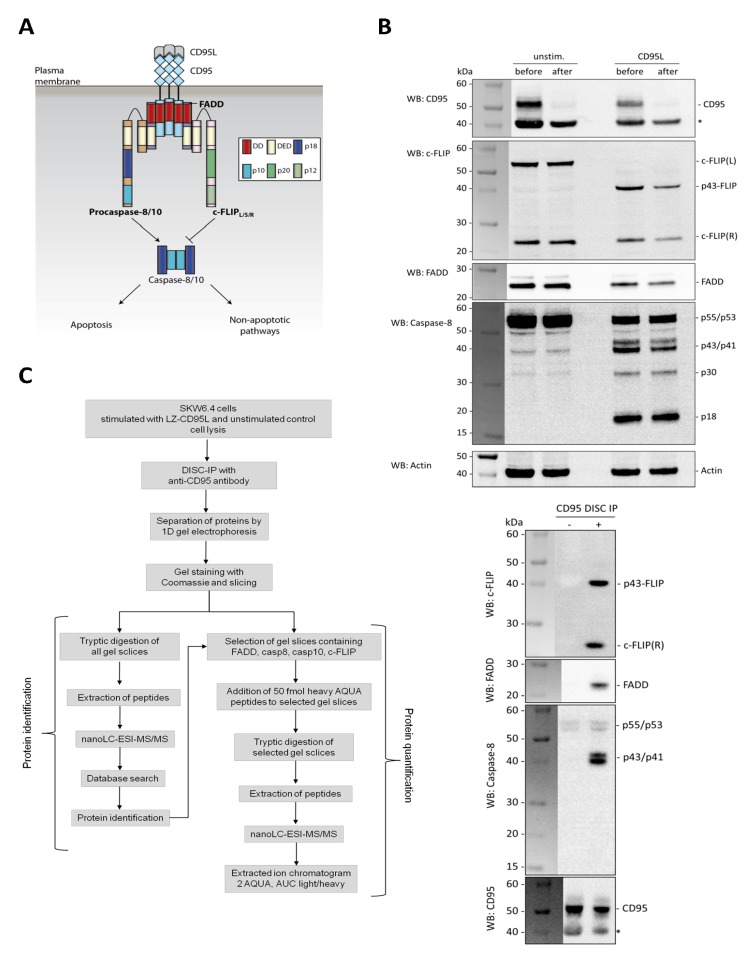
Quantitative immunoprecipitation of the CD95 DISC and workflow of the experiments. (**A**) Classical model of the CD95 DISC. The DISC is formed after CD95L stimulation and consists at least of the adapter protein FADD, procaspase-8, procaspase-10 and c-FLIPs. FADD is recruited to CD95 through death domain (DD) interactions. Subsequently, procaspases-8 and -10 and c-FLIPs are recruited to FADD through death effector domain (DED) interactions. The classical model of the DISC assumes a one-to-one interaction between FADD and procaspases. (**B**) Western blot analysis of total lysates and DISC IPs. 5 × 10^7^ SKW6.4 cells were stimulated with 1 µg/mL LZ-CD95L for 15 min or left untreated. Western blot (WB) analysis of the total cell lysates were performed before and after immunoprecipitation with 10 μg of anti-CD95 antibody. * loading control: actin. The signal for CD95, which is clearly observed in the lysates, completely disappeared after IP. The bands marked with an asterisk are unspecific. The amounts of procaspase-8a/b, procaspase8a/b cleavage products (p43/p41, p30 and p18); c-FLIP isoforms (c-FLIP_L/R_)/cleavage products (p43-FLIP) and FADD were also reduced in the lysates after DISC IP. Therefore, it is important to perform an IP prior to the quantification of the DISC proteins as shown below. The IPs demonstrate specific recruitment of FADD, c-FLIP and procaspase-8 to the CD95 DISC only upon LZ-CD95L stimulation. (**C**) Quantification workflow. DISC IPs from stimulated or unstimulated SKW6.4 cells were separated by 1D gel electrophoresis and analyzed by mass spectrometry. Proteins in the IPs were first identified by database search. Selected regions of the gel containing the DISC proteins were further processed for quantification. Heavy AQUA peptides were spiked in, and heavy to light ratios were calculated from extracted ion chromatograms of the individual peptide pairs.

Recently, we studied the stoichiometry of the CD95 death-inducing signaling complex (DISC) by biochemical isolation followed by quantification using AQUA-peptide based mass spectrometry [1]. The DISC is formed after stimulation of the death receptor CD95 resulting in apoptosis initiation (Figure 1A). Apoptosis plays a fundamental role in the development and homeostasis of multicellular organisms and deregulation of apoptosis can lead to several diseases, such as autoimmune diseases, neurodegenerative diseases or cancer. Apoptosis can be induced by extra- as well as intracellular stimuli. Extracellular signals trigger apoptosis *via* the induction of death receptors (DR) on the plasma membrane. CD95 (also named Fas/APO-1) belongs to the best characterized DRs, together with TNFR1, TRAIL receptor 1 (TRAIL-R1) and TRAIL-R2 [8,9,10]. Other DRs include DR3 and DR6, EDA-R and NGF-R [9,10,11]. The CD95-induced apoptotic pathway is one of the best-studied signaling pathways. CD95 can be activated by its natural ligand CD95L or agonistic antibodies, such as anti-APO-1 [12]. Stimulation induces the oligomerization of receptors and formation of the DISC. The DISC consists of the adaptor protein FADD, the initiator caspases procaspase-8 and -10 and cellular FLICE inhibitory proteins (c-FLIPs). The molecular interactions in this complex are mediated by homotypic interaction motifs. Procaspase-8 is present at the DISC in two isoforms: procaspase-8a/b (p55/p53). Both have two death effector domains (DEDs) in their N-terminal prodomain, which are required for its recruitment into the DISC. Furthermore, it contains a large (~20 kDa) and a small (~10 kDa) catalytic subunit [13]. Procaspase-8 is activated at the DISC by dimerization and subsequent cleavage [13] (Figure 1A). Procaspase-8a/b processing at the DISC generates the cleavage products p43/p41, p30, p26/p24, p18 and p10 and is regulated by c-FLIPs [14]. Active caspase-8 constitutes a heterotetramer consisting of two large (p18) and two small (p10) catalytic subunits and can directly cleave the effector caspases-3 and -7, which then cleave a variety of substrates eventually resulting in cell death [15,16]. Furthermore, caspase-8 can cleave the pro-apoptotic protein Bid, generating tBid, which then translocates to the mitochondria where it triggers mitochondrial outer membrane permeabilization (MOMP) and the release of pro-apoptotic factors into the cytosol, such as cytochrome c and Apaf-1 [16,17]. Subsequently another complex, termed apoptosome, is formed that is required for the activation of procaspase-9. Caspase-9 also cleaves and activates procaspases-3 and -7 and thereby amplifies the extrinsic signal. 

Here we describe our strategy of investigating the stoichiometry of the CD95 DISC using biochemical pull-down and quantification by AQUA peptide-based quantitative mass spectrometry. The biological implications of this analysis were reported by us in [1]; however, below we elaborately describe the methodology of this analysis. We discuss in detail the selection and validation of AQUA peptides and present a workflow which can be applied to the analysis of protein complexes in general.

## 2. Experimental Section

### 2.1. Cell Lines & Antibodies

B lymphoblastoid SKW6.4 cells were cultured in RPMI (Life Technologies, Germany), 10 mM HEPES (Life Technologies, Germany), 50 µg/mL gentamycin (Life Technologies, Germany), supplemented with 10% fetal calf serum (Life Technologies, Germany) in 5% CO_2_.

The anti–caspase-8 monoclonal antibody C15 (mouse IgG-2b) recognizes the p18 subunit of caspase-8 [18]. The anti-FLIP monoclonal antibody NF6 (mouse IgG-1) recognizes the N-terminal part of c-FLIP [19]. The anti-FADD monoclonal antibody 1C4 (mouse IgG-1) recognizes the C-terminal part of FADD [20]. The actin antibody was purchased from Sigma-Aldrich. The CD95 antibody C-20 for Western blot detection was obtained from Santa Cruz Biotechnology. Anti–APO-1 (anti-CD95) is an agonistic monoclonal antibody (IgG-3), which recognizes an epitope in the extracellular part of CD95 [12]. Horseradish peroxidase–conjugated goat anti–mouse IgG-1, -2a, and -2b antibodies were from Southern Biotech, the goat anti-rabbit antibody was from Santa Cruz Biotechnology. The coding sequence of LZ-CD95L [21] was cloned into a pIRESpuro3 plasmid (Takara Bio Inc.). Recombinant LZ-CD95L was produced using 293T cells that were stably transfected with this vector. All chemicals used were of analytical grade and purchased from Merck or Sigma-Aldrich.

### 2.2. Sample Preparation for AQUA-Peptide Validation

Heavy labeled AQUA peptides and their unlabeled (light) counterparts exactly quantified by amino acid analysis were acquired from Thermo-Fisher (Ulm, Germany) and delivered in aliquots with concentrations of (5.0 ± 5%) pmol/µL except the unlabeled AQUA peptide NLYDIGEQLDSEDLASLK, which was delivered in a concentration of (2.3 ± 5%) pmol/µL. Quantity check of AQUA peptides was performed by mixing heavy and light peptides in equal amounts prior to dilution with 50% acetonitrile (ACN)/ 50% H_2_O/0.1% formic acid (FA) to a final concentration of 1 pmol/µL. Offline MS1 spectra were acquired on a QTof Ultima mass spectrometer (Waters GmbH, Eschborn, Germany). Basic calibrations were acquired applying dilution series of the light AQUA peptide in a range from 1-200 fmol spiked with 50 fmol of the corresponding heavy AQUA peptide. NanoESI-LC MS1 analysis was performed using a nanoAcquity UPLC system (Waters GmbH, Eschborn, Germany) on an LTQ-Orbitrap XL mass spectrometer (Thermo Scientific, Bremen, Germany) by applying stepped linear gradients over one hour from 5–80% ACN in H_2_O/0.1% FA with a resolution of 60,000 at *m/z* 400. 

### 2.3. Sample Preparation for Protein Identification and Protein Quantification

Protein separation and in-gel digestion were carried out as described before [1]. For protein identification all gel slices were analyzed. For protein identification only selected gel slices containing FADD, caspase-8, caspase-10 and c-FLIP and their cleavage products were processed. 

### 2.4. Mass Spectrometry

Tryptic peptides were separated using a nanoAcquity UPLC system (Waters GmbH, Eschborn, Germany). Peptides were loaded on a C18 trap column (180 µm × 20 mm) with a particle size of 5 µm (Waters GmbH, Eschborn, Germany). Liquid chromatography separation was performed on a BEH130 C18 main- column (100 µm × 100 mm) with a particle size of 1.7 µm (Waters GmbH, Eschborn, Germany) at a flow rate of 0.4 µL/min. For protein identification a 1h gradient was applied and set up as follows: from 0–4% B in 1 min, from 4–40% B in 39 min, from 40–60% B in 5 min, from 60–85% B in 0.1 min, 6 min at 85% B, from 85–0% B in 0.1 min, and 9 min at 0%. For protein quantification the following 2 h gradient was applied: from 0–4% B in 1 min, from 4–30% in 79 min, from 30–45% B in 10 min followed by an increase in 10 min of solvent B from 45–90% and for a further 10 min at 90% solvent B. After this step the concentration was stepped down to 0% solvent B and continued for 15 min. Solvent A contained 98.9% water, 1% acetonitrile and 0.1 % formic acid, solvent B contained 99.9% acetonitrile and 0.1% formic acid. The nanoUPLC system was coupled online to an LTQ Orbitrap XL mass spectrometer (Thermo Scientific, Bremen, Germany). Data were acquired by scan cycles of one FTMS scan with a resolution of 60000 at *m/z* 400 and a range from 300–2000 *m/z* in parallel with six MS/MS scans in the ion trap of the most abundant precursor ions. Instrument control, data acquisition and peak integration were performed using the Xcalibur software 2.1 (Thermo Scientific, Bremen, Germany).

Database searches were performed against the human NCBInr database using the MASCOT search engine (Matrix Science, London, UK; version 2.2.2). Peptide mass tolerance for database searches was set to 5 ppm and fragment mass tolerance was set to 0.4 Da. Significance threshold was *p* < 0.01. Carbamidomethylation of cysteine was set as a fixed modification. Variable modifications included oxidation of methionine and deamidation of asparagine and glutamine. One missed cleavage site in case of incomplete trypsin hydrolysis was allowed. 

Extracted ion chromatograms derived from exact Orbitrap mass scans from each AQUA/target peptide pair were generated. Areas under curves were obtained by manual baseline determination and integration of the peak area of the light and heavy peptide, respectively. The heavy to light ratio of each AQUA peptide pair was calculated. Finally, since the absolute amount of heavy peptide was 50 fmol in all experiments the total amount of light peptide could be determined from the H/L ratios. In this way the total amount of the corresponding protein in each gel slice was obtained. The overall amount for each protein was calculated by summing up all amounts in the different gel slices. 

## 3. Results

### 3.1. Identification of Proteins from CD95 Immunoprecipitation (IP) Experiments

The first step in our quantification workflow was to identify proteins that were associated with the DISC. B lymphoblastoid SKW6.4 cells were stimulated with leucine zipper CD95 ligand (LZ-CD95L) or left untreated as control. Cells were lysed under mild conditions to maintain the integrity of the DISC. Subsequently, the complex was immunoprecipitated with anti-CD95 antibody and co-precipitated proteins were eluted from the Protein-A-Sepharose beads. Importantly, in conditions used we could co-immunoprecipitate almost 100% of CD95 present in the cells and therefore pull- down all CD95 DISCs formed (Figure 1B). Indeed, using Western blot we detected in the IP: FADD, procaspase-8 and c-FLIP as well as their cleavage products (Figure 1B). To identify proteins from the IPs, we initially analyzed the complete protein mixtures. Proteins were reduced, alkylated at cysteine residues and digested with trypsin in solution. Mass spectrometric analysis was performed on an LTQ Orbitrap mass spectrometer coupled online to a nanoUPLC system. Proteins were identified by database search using the MASCOT search algorithm. However, with this approach only procaspase-8 could be unambiguously identified in the IPs. The other key components which were already known from previous studies to be present in the complex, namely FADD and c-FLIP, were not detected, probably due to the high complexity of the protein mixture. Therefore, we separated the proteins by one-dimensional polyacrylamide gel electrophoresis (1D-PAGE) under denaturing conditions prior to digestion to reduce the complexity. After staining with sensitive Coomassie, the entire gel was sliced into 28 pieces and each gel piece was individually processed. After reduction and alkylation, proteins were proteolytically digested in-gel with trypsin. Tryptic peptides were extracted from the gel slices and analyzed by nanoLC-ESI-MS/MS as described above. The identification workflow is illustrated in Figure 1C, left panel. In total, more than 1000 proteins were identified in the DISC IPs. In addition to the known components of the DISC some of these hits might represent not yet characterized DISC-associated proteins. For further quantification of proteins in the CD95 DISC IPs, only slices containing the major DISC proteins, namely procaspase-8 and -10, FADD, and c-FLIP and their cleavage products were analyzed. 

### 3.2. Quantification of Major Protein Components in the DISC

In order to elucidate the stoichiometry of proteins in the DISC the major protein components FADD, procaspase-8 and -10, and c-FLIP needed to be quantified. To this end we employed the AQUA strategy, a recently described method for absolute quantification of proteins [4]. For each protein two to three heavy labeled AQUA standards were selected and chemically synthesized.

### 3.3. Design of AQUA Peptides

The selection of AQUA peptides for quantification is based on several rules [22]. From the chemical point of view, AQUA peptides should be stable under all treatment conditions used for proteolytic digestion and mass spectrometry. Amino acid residues which can easily oxidize such as methionine, cysteine and tryptophan should be excluded. N-terminal glutamine residues can undergo pyroglutamate formation to a certain extent and should therefore be avoided. Likewise, sequences with labile amide bonds such as the Asp-Pro bond can undergo hydrolysis and should not be selected. 

From the mass spectrometry point of view peptides should be synthesized in such a way, that the isotopic clusters in the mass spectra do not overlap. We have incorporated either [^13^C_6_,^15^N_2_] lysine or [^13^C_6_,^15^N_4_] arginine at the C-termini of the selected heavy AQUA peptides thus resulting in a mass increase of 8 and 10 Da, respectively, for the singly charged molecules in relation to their light counterparts.

**Figure 2:**
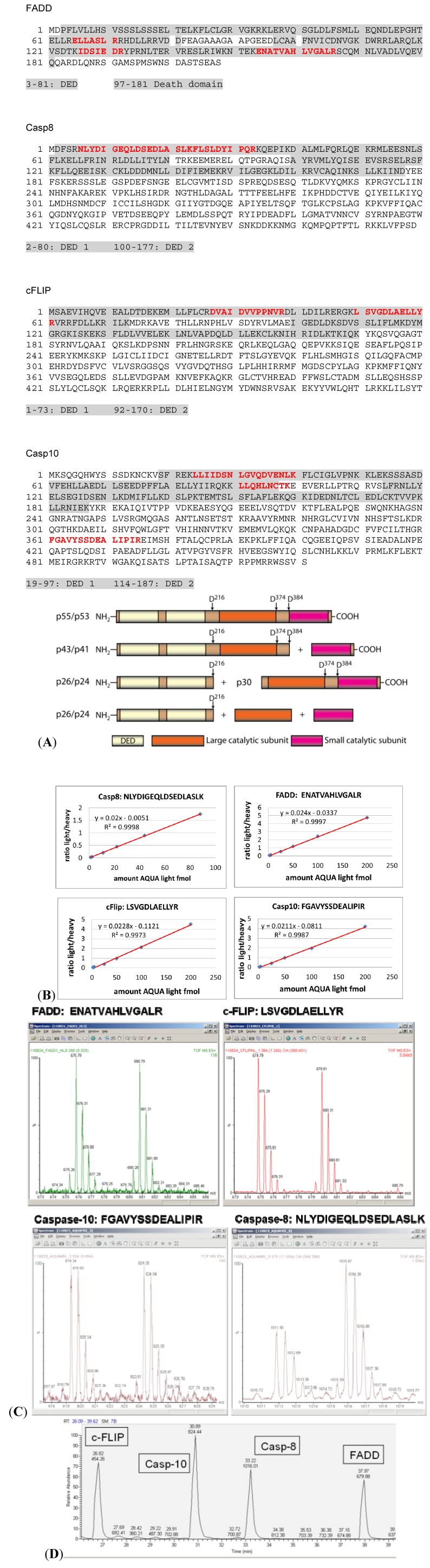
Selection and validation of AQUA peptides. (**A**) Amino acid sequences of the major components FADD, procaspase-8/10 and c-FLIP of the CD95 DISC. Selected AQUA peptides are highlighted in red and DED and DD are marked in grey. The scheme of procaspase-8a/b processing is shown below. (**B**) Basic calibration of corresponding light and heavy AQUA peptide pairs. Variable amounts of the light peptide ranging from 1 to 200 fmol were added to a constant amount of 50 fmol of the corresponding heavy peptide. All AQUA peptide pairs showed linearity in this concentration range when analyzed by mass spectrometry on an LTQ Orbitrap instrument. (**C**) Quality control of AQUA peptides. Light and heavy AQUA peptides pairs were mixed in a 1:1 ratio and analyzed by mass spectrometry on a QToF instrument. Heavy to light ratios were close to 1:1 or 1:2 confirming the specified peptide concentrations. (**D**) Chromatographic behavior of AQUA peptides. Extracted ion-chromatogram of heavy and light AQUA peptides are shown for c-FLIP, FADD, procaspase-8, and procaspase-10. Heavy and light peptides were mixed and analyzed by nanoLC-ESI-MS1 on an Orbitrap mass spectrometer.

For a reliable quantification, proteins should ideally be represented by at least two different peptides. In the case of FADD, three peptides were selected, spanning amino acid (aa) 65–71, aa 126–132 and aa 154–166, respectively (Figure 2A). The first peptide is located within the death effector domain (DED); the other two peptides are within the death domain (DD). In our previous mass spectrometric analysis FADD was detected in only one single band at a molecular weight range between 25–28 kDa. Furthermore, phosphorylation of FADD at Ser 194 has been observed [20] as well as cleavage at the C-terminal Asp201 (unpublished observations). However, since neither phosphorylation nor cleavage is affecting the region of the selected AQUA peptides they should give comparable results.

Whereas FADD was found at its expected molecular weight in the 1D gel caspase-8 and caspase-10 as well as c-FLIP were detected in gel slices corresponding not only to their respective molecular weights in the mass range of 38–60 kDa, but also in the lower mass ranges of 20–35 kDa. The additional bands at lower molecular weights represent the different cleavage products of these proteins. This had to be considered in the selection of suitable AQUA peptides. Procaspase-8a/b undergoes processing at the DISC yielding N-terminal procaspase-8a/b prodomains (p26/p24), which remain bound to the DISC [23] (Figure 2A, lower part). Therefore, two peptides within the prodomain covering aa 6–23 and aa 24–33 were selected for quantification and were synthesized as heavy AQUA standards (Figure 2A). 

For procaspase-10 we selected three peptides, two of which were located within DED and one within the subunit p23/p17 (Figure 2A).

c-FLIP_L_ is cleaved to p43-FLIP and only this part remains at the DISC. In addition, there are two short c-FLIP isoforms reported: Short (c-FLIP_S_, 26 kDa) and Raji (c-FLIP_R_, 25 kDa) that are bound to the DISC also *via* DED interactions [9]. In SKW6.4 cells c-FLIP_R_ is the only short isoform detected. Therefore, we chose two AQUA peptides from the first DED, namely aa 27–38 and aa 50–62 (Figure 2A).

Finally, it is essential that the chosen peptide is unique for the protein of interest. Therefore, all selected peptides were analyzed using the BLAST algorithm to avoid any interference or overlap with other protein sequences.

In total, 10 heavy AQUA peptides were chemically synthesized. Details of all heavy and light peptide pairs are summarized in Table 1.

**Table 1:** Sequences of AQUA peptides. Heavy peptides are labeled either with [^13^C_6_,^15^N_2_] lysine or with [^13^C_6_,^15^N_4_] arginine at their respective C-terminus.

Protein	Sequence	Light	Heavy
		*m/z* (charge state)	*m/z* (charge state)
FADD	ENATVAHLVGALR	675.8781 (2+)	680.8822 (2+)
		450.9211 (3+)	454.2572 (3+)
	IDSIEDR	424.2114 (2+)	429.2156 (2+)
	ELLASLR	401.2451 (2+)	406.2492 (2+)
Caspase-8	NLYDIGEQLDSEDLASLK	1011.9969 (2+)	1016.0040 (2+)
	FLSLDYIPQR	626.3402 (2+)	631.3444 (2+)
c-FLIP	LSVGDLAELLYR	674.8772 (2+)	679.8813 (2+)
	DVAIDVVPPNVR	647.3617 (2+)	652.3658 (2+)
Caspase-10	LLIIDSNLGVQDVENLK	942.0279 (2+)	946.0350 (2+)
	LLQHLNCTK	563.8055 (2+)	567.8126 (2+)
	FGAVYSSDEALIPIR	819.4303 (2+)	824.4345 (2+)

### 3.4. Validation of AQUA Peptides

Although the non-labeled “light” AQUA peptides are not necessarily required for the quantification itself they are valuable tools for basic calibration purposes as well as for the determination of the detection limit and sensitivity. To test the linearity of the quantification in the expected concentration range and the recovery rate, we used 50 fmol of the heavy peptide and added variable amounts of the light counterpart. This is exemplified for one peptide each of FADD, procaspase-8, procaspase-10 and c-FLIP (Figure 2B). Sequences of the four selected AQUA peptides are indicated in Figure 2B. The calibration curves showed good linearity in the range from 1 to 200 fmol of spiked-in light AQUA peptides (Figure 2B). The detection limit of AQUA peptides was about 1 fmol for Orbitrap quantifications.

To test the quality of both, light and heavy AQUA peptides as supplied by the vendor, stock solutions were mixed in a 1:1 (v:v) ratio. The sequences of the peptide pairs were the same as above for the calibration curves. Mass spectrometric analysis of those mixtures was performed on a QToF instrument operated off-line in the MS1 modus (Figure 2C). As illustrated in Figure 2C the MS1 traces of peptide mixtures showed a very good 1:1 ratio of the m/z signals for the doubly protonated parent ions of FADD, c-FLIP and procaspase-10 and a 1:2 ratio for the m/z signals of procaspase-8. These analyses were repeatedly performed over a period of time to test whether the stock solutions changed in concentration due to adsorption or degradation. 

Another important issue is the knowledge of chromatographic behavior of the selected peptides. Heavy and light AQUA peptides derived from FADD, c-FLIP, procaspase-8 and -10 were mixed and separated on a UPLC column using a linear one hour gradient of acetonitrile in 0.1% formic acid. The heavy and light peptide pairs were the same as used before. Figure 2D shows the extracted ion chromatogram for c-FLIP, procaspase-10, procaspase-8 and FADD. All four peptide pairs were well separated and eluted in sharp and symmetric peaks. Importantly, the retention times for corresponding light and heavy peptide pairs were essentially the same. No retention time shifts were observed which can occur when deuterated peptides are used as heavy AQUA standards [22].

### 3.5. Quantification Workflow

Our study mainly focused on the investigation of the stoichiometry of the DISC proteins FADD, c-FLIP, procaspase-8 and -10. Below we describe the quantification workflow in detail, though it has to be noted that the biological implications of these findings were reported in [1]. Based on our previous results obtained from the identification workflow, we already knew the gel slices which contained our target molecules. Only these slices were included in the quantification workflow (Figure 1C, right panel). FADD was detected only once in gel slices corresponding to a molecular weight of 25–28 kDa. In contrast, the three DED proteins procaspase-8, procaspase-10 and c-FLIP are further processed at the DISC and in addition have isoforms different in their molecular masses. They were detected in gel slices according to a mass range of 38–60 kDa, but also in gel slices corresponding to a lower molecular weight around 20–35 kDa.

After reduction and alkylation of proteins in the gel slices and several washing steps, the selected gel slices were spiked with 50 fmol of each heavy AQUA peptide prior to tryptic digestion. Thus, all 10 heavy peptides were present in each sample. Although all AQUA peptides used in our study represent tryptic peptides *per se* and cannot be further digested it is advisable to add them as early as possible in the workflow to minimize sample to sample variation. After incubation with trypsin, peptides were extracted from the gel pieces and analyzed by nanoLC-ESI-MS/MS. 

Quantification of the proteins was achieved in several steps. Firstly, m/z values for the doubly and triply-charged ions of all 10 light and heavy AQUA peptides were calculated *in silico*. These *m/z* values were listed in an inclusion table. Secondly, during mass spectrometric analysis on the Orbitrap, the MS1 trace was recorded from all peptides above the detection limit derived from all proteins present in the corresponding gel slice. In contrast, the MS2 trace was only acquired from peptides specified in the inclusion table and reaching the data dependent acquisition threshold. The MS2 trace was later used for a correct assignment of the peptides. Thirdly, extracted ion chromatograms for the selected light and heavy peptide pairs were generated from the MS1 trace. Finally, the area under the curve (marked area, MA) was obtained by manual integration of the peak area of the light and heavy peptide, respectively. Since the spiked-in amount of the heavy peptide was known to be 50 fmol, the total amount of the corresponding light peptide could be calculated based on the light to heavy ratio. 

**Figure 3:**
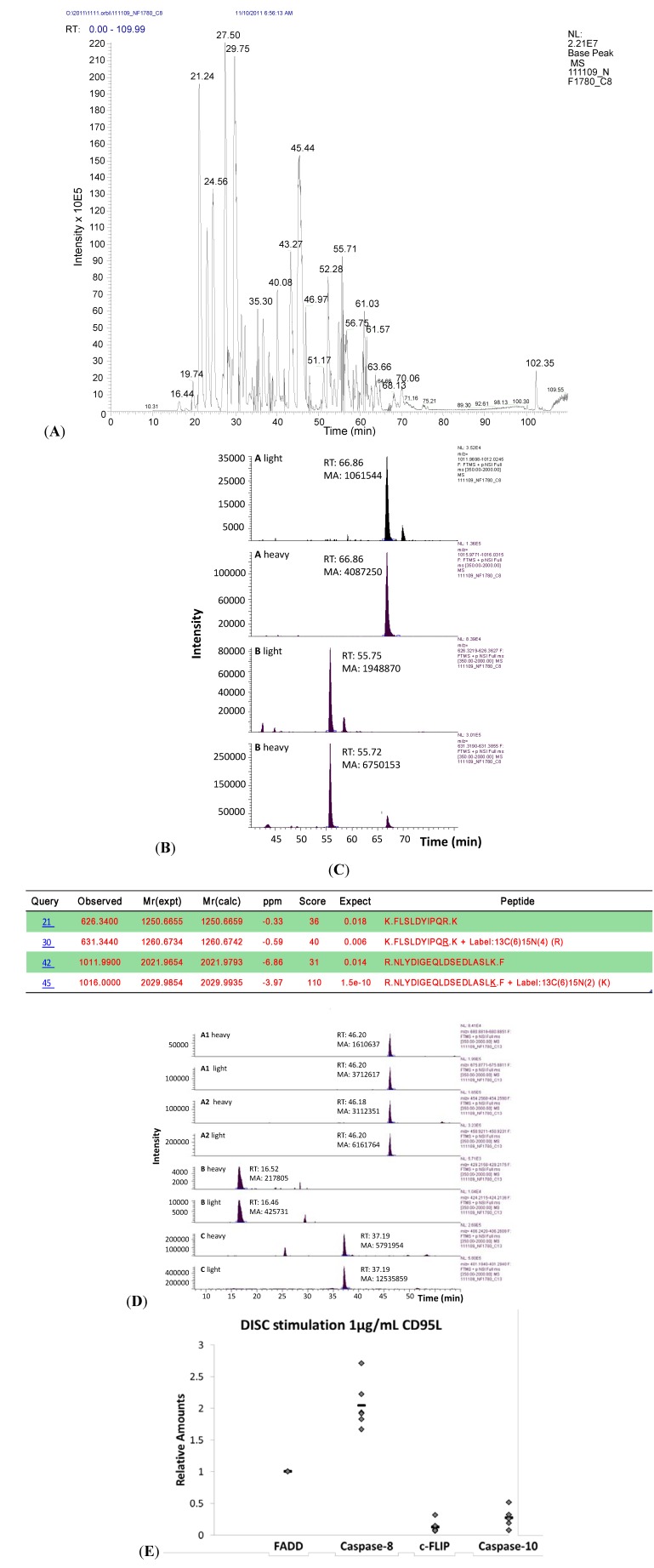
Quantification of the DISC. (**A**–**C**) Workflow for the quantification as exemplified for caspase-8. (**A**) Base peak chromatogram of the mass spectrometric analysis of gel slice 8. A two hour gradient was applied. (**B**) Extracted ion chromatograms of the endogenous light and spiked-in heavy peptide NLYDIGEQLDSEDLASLK (A light and A heavy) and endogenous light and spiked-in heavy peptide FLSLDYIPQR. (B light and B heavy). RT: retention time; MA: marked area (AUC). The ratios of light and heavy areas are calculated and used for quantification of the light peptides. (**C**) Database search of MS2 data confirmed the sequence of the two light and heavy peptide pairs of procaspase-8. (**D**) Extracted ion chromatograms of corresponding peptide pairs for FADD in gel slice 13. A1 heavy/light and A2 heavy/light represent the same peptide sequence in two different charge states, 2+ and 3+, respectively, with the same retention time of 46 min. The second (B heavy/light) and third peptide (C heavy/light) are more hydrophilic and elute earlier from the reverse phase column. (**E**) Stoichiometry of the CD95 DISC. Total amounts of proteins were calculated from the extracted ion chromatograms and normalized to FADD. The stoichiometry was determined to be 1:2:0.1:0.3 for FADD, procaspase-8, c-FLIP and procaspase-10.

An example of this workflow is given in Figure 3A–C for procaspase-8. The first panel (Figure 3A) shows the base peak chromatogram for gel slice 8 and illustrates the high complexity of the sample. Extracted ion chromatograms for two different AQUA peptide pairs derived from procaspase-8 are shown in Figure 3B. The first two chromatograms in Figure 3B (A light and A heavy) represent the light and heavy peptide pairs with the sequence NLYDIGEQLDSEDLASLK and a retention time (RT) of about 66.9 min. The marked area (MA) of the heavy peptide corresponds to 50 fmol and is 3.85 times larger than the MA of the light peptide. Thus, the total amount of light peptide in this gel slice is about 14 fmol. The last two chromatograms in Figure 3B (B light and B heavy) show the extracted ion chromatograms of the light and heavy peptide pairs FLSLDYIPQR with a RT of 55.7 min. The heavy to light ratio calculated from the MA is 3.46 which corresponds to 14.5 fmol of light peptide. This result is in good accordance with the first measurement using the first heavy peptide.

To check whether the extraction ion chromatograms represent indeed the two peptide pairs, we performed a database search using the MS2 data. The search result is summarized in Figure 3C. Both peptides in their light and heavy form were unambiguously identified in the mass spectrometric analysis of gel slice 8, thus confirming that our assignment was correct.

In total, six DISC IPs were performed. Procaspase-8a/b was detected in gel slices 5–15. However, the amount of procaspase-8a/b varied strongly in the different gel slices due to its cleavage to p43/p41, p26/p24, p30 and p18/p10. As can be seen from Table 2 we observed two peaks of caspase-8, one in gel slices 6–9 and a second one in gel slices 13 and 14. In contrast, gel slices 10–12 contained almost no caspase-8. Since gel slices 6–9 correspond to a molecular weight of about 38–60 kDa we assume that these slices contain unprocessed procaspase-8 and the cleavage product p43/p41. Gel slices 13 and 14 correspond to a molecular weight region around 30 kDa and contain the processed procaspase-8a/b prodomains which are known to remain at the DISC. 

**Table 2:** Quantification of caspase-8 in different gel slices and total amounts in the six DISC IPs.

	IP 1	IP 2	IP 3	IP 4	IP 5	IP 6
Gel slice	fmol					
**5**	n.d.	2	3	4	12	n.d.
**6**	12	22	37	37	51	n.d.
**7**	34	83	8	23	58	8
**8**	7	14	7	28	26	8
**9**	10	22	7	48	47	15
**10**	n.d.	1	n.d.	2	2	1
**11**	n.d.	1	n.d.	1	2	1
**12**	2	4	6	8	6	4
**13**	21	54	8	55	45	33
**14**	7	14	1	51	44	15
**15**	n.d.	n.d.	n.d.	1	1	n.d.
						
**Total Caspase-8**	91	217	76	259	293	84

FADD was quantified using three different peptides as depicted in Table 1. The extracted ion chromatograms for all three peptides taken from the mass spectrometry analysis of gel slice 13 are depicted in Figure 3D. Peptide ENATVAHLVGALR was detected in two different charge states. The A1 heavy/light chromatograms represent the charge state 2+ whereas the A2 heavy/light chromatograms represent the charge state 3+. The retention time is about 46 min and the heavy to light (H/L) ratio is calculated as 0.433 and 0.505, respectively, corresponding to 115 and 99 fmol of FADD in gel slice 13. The second FADD peptide IDSIEDR shown in Figure 3D (B heavy and light) is the most hydrophilic one and eluted already at 16.5 min. The H/L ratio for this peptide is 0.512 corresponding to a total amount of 98 fmol of FADD in gel slice 13. The third peptide used for quantification of FADD was ELLASLR (Figure 3D; C heavy and light). The H/L ratio was 0.46 corresponding to 108 fmol of FADD.

For procaspase-10 and c-FLIP the same procedure was applied. Two AQUA peptides were selected for c-FLIP and three AQUA peptides for procaspase-10 (Table 1). For c-FLIP we could not detect the second AQUA peptide DVAIDVVPPNVR in all DISC IPs. In those cases quantification was based on one peptide only. For procaspase-10 the first two peptides in Table 1 were derived from the DED whereas peptide FGAVYSSDEALIPIR was selected from the subunit p23/p17. Since procaspase-10 is also further processed at the DISC we could detect the latter peptide only in the gel slices corresponding to the intact protein.

In summary, six replicate DISC IPs were carried out from 5x10^7^ SKW6.4 cells stimulated with 1 μg/mL LZ-CD95L. Table 3 summarizes the results of the quantification of the four DISC proteins. Total amounts of the different DISC proteins were obtained by adding up the amounts in the individual gel slices. Although the total amounts of DISC proteins varied between the different DISC IPs, the ratios of the respective DED proteins to FADD were very reproducible. This, in turn, means that although the total amount of DISC which was immunoprecipitated differed in the six experiments, the stoichiometry of the complex did not change. 

**Table 3:** Absolute amounts of FADD, procaspase-8, c-FLIP and procaspase-10 detected in DISC IPs after stimulation of SKW6.4 cells with 1 µg/mL LZ-CD95L. The ratios of DISC proteins normalized to FADD are given below.

		IP 1	IP 2	IP 3	IP 4	IP 5	IP 6	Mean	SD
**FADD**	fmol	134	132	46	34	113	46		
**Procaspase-8**	fmol	259	293	84	91	217	76		
**c-FLIP**	fmol	9	11	3	5	8	14		
**Procaspase-10**	fmol	25	36	12	11	58	3		
**Procaspase-8/FADD**		1.9	2.2	1.8	2.7	1.9	1.7	2.0	0.4
**c-FLIP/FADD**		0.1	0.1	0.1	0.1	0.1	0.3	0.1	0.1
**Procaspase-10/FADD**		0.2	0.3	0.3	0.3	0.5	0.1	0.3	0.1

To further illustrate the method described above we have obtained the following ratios of the proteins: Overall, a mean ratio of 2.0 was measured for procaspase-8 to FADD. For c-FLIP to FADD we calculated a ratio of 0.1 and for procaspase-10 to FADD a ratio of 0.3. As illustrated in Figure 3E, our results determined a stoichiometry of 1:2:0.1:0.3 for FADD, procaspase-8, c-FLIP and procaspase-10 in the CD95 DISC. These data have been reported in [1] and served as a basis for the development of the new model of procaspase-8 activation and stoichiometry of the DISC. This stoichiometry provided new insights into DISC organization and apoptosis induction [1]. 

## 4. Discussion

We aimed to develop an approach for the elucidation of the stoichiometry of the CD95 DISC by quantitative mass spectrometry. Although numerous proteins linked to the DISC were detected in our IPs, we focused on those proteins that were already known to be important members of the DISC, namely the adaptor protein FADD, procaspase-8 and -10 and c-FLIP. To analyze the DISC stoichiometry, we made use of the AQUA technique as a reliable and sensitive method for the determination of absolute amounts of proteins in complex mixtures.

Mostly, quantifications using the AQUA technique are performed by a gel-free approach. In our study we used a separation of the protein samples by one-dimensional polyacrylamide gel electrophoresis (1D-PAGE). In this way, we could reduce sample complexity and separate different isoforms of DISC proteins and their cleavage products. Thus, we can state that this methodology is important when analyzing the composition of apoptotic complexes with analytes related to multiple cleavage products. 

The sequences of AQUA peptides were carefully selected according to the accepted standards, which we believe is a key step for the successful quantitative analysis. Importantly, the sequences of peptides were unique for the protein of interest and we avoided amino acids that could easily be chemically altered by oxidation or hydrolysis. Nevertheless, a basic validation step of the AQUA peptides is essential for the further quantitative analysis. To this end, we used the combination of light and heavy AQUA peptides and obtained linear calibration functions upon titration with different amounts of the peptides and determined the limit of detection for the individual AQUA peptides. It has to be noted that typically protein quantification with AQUA peptides is achieved using only heavy AQUA peptides. Here we show that the additional calibration step with the light AQUA peptide is essential for the successful validation of the quantification workflow. Therefore, we state that only AQUA peptides that could be validated in this way are suitable for the quantification analysis.

Another important point of the analysis is the application of several AQUA peptides for the quantification of each protein. This provides reliable quantification of the different proteins. As it has to be noted, even ideal AQUA peptides might not provide reliable information on the stoichiometry due to some peculiarities of its chemical structure and unforeseen interactions

Interestingly, the total amounts of DISC proteins varied between the different experiments, but the stoichiometry, e.g., ratio between procaspase-8 and FADD, remained unaltered. We considered that we might have some variations in the final quantities of the complex that might derive from the sample preparation, though we always used the same amount of cells, antibodies, protein beads, time for the immunoprecipitation. Thus, this question requires further clarification and analysis. 

Another very important point to address for selecting the AQUA peptides is whether the corresponding protein region remains unmodified in the complex. The latter is especially significant for apoptotic complexes, where cleavage is a major protein modification taking place. Based on the knowledge about the CD95 DISC, we selected AQUA peptides corresponding to the regions of DEDs/DDs of procaspase-8/10, c-FLIP and FADD, respectively. This certainly provides a number of limitations. Namely, procaspase-8 is typically cleaved at the DISC and N-terminal prodomains remain bound to the DISC [23]. Thus, using AQUA peptides we could estimate only N-terminal cleavage products of procaspase-8 containing the prodomain. With these AQUA peptides it is not possible to measure the amount of C-terminal procaspase-8 cleavage products bound to the complex if we would assume they remain at the DISC. However, according to our previous Western blot analysis of the complex, only 1–5% of C-terminal cleavage products, *i.e.*, p10, p18 and p30, remain bound to the DISC [14,24], therefore, this should not influence our analysis significantly. In addition, using AQUA peptides against N-terminal prodomains we also could not quantify prodomains if they would have been cleaved in the course of apoptosis and dissociate from the DISC. In our extensive experience we have never observed significant cleavage at the DEDs of N-terminal procaspase-8/10 prodomains using Western blots. This permits us to assume that the prodomain stays largely intact at this complex and does not undergo cleavage. Furthermore, we also never observed the cleavage at the DEDs or DDs of FADD except for a small C-terminal fragment (data not shown). Therefore, AQUA peptides selected against DED/DD of FADD should provide reliable information about its presence in the complex. However, cleavage certainly has to be considered for the analysis of other apoptotic complexes, where it might significantly influence the quantitative readout using AQUA peptides selected against cleaved parts of the complex.

Interestingly, independently from our study, Dickens *et al.* [25] developed a label-free mass spectrometry approach for the analysis of the DISC formed at the TRAIL-R. Surprisingly, they also discovered that procaspase-8 outnumbers FADD at the TRAIL-R complex that allows suggesting that chain formation is a general paradigm for procaspase-8 activation in extrinsic apoptosis. Thus, these two independent mass spectrometry approaches provided comparable data and in this way both methods proved to be a valuable tool for the analysis of the high molecular weight platforms.

## 5. Conclusions

In this study we have presented a mass spectrometry workflow that can be implemented for the analysis of the stoichiometry of any multiprotein complex. We have shown it on the example of the CD95 DISC protein quantification where we have applied the AQUA standard peptide quantification technique. We have demonstrated how our modification of AQUA peptide approach could be successfully used for the analysis of the stoichiometry of the death-inducing signaling complex. The knowledge of the stoichiometry of the high molecular weight platform is a key to the understanding of the signaling events mediated by the particular platform. Furthermore, systems biology is based on the quantitative assessment of the interactions in the signaling network. The quantification of proteins in complexes and the determination of their stoichiometry employing mass spectrometry techniques have an important impact that is essential for the further development of the field and application of these findings towards signaling pathways connected to various diseases.

## Abbreviations

IP: (Immunoprecipitation)
DISC: (death-inducing signaling complex)
DR: (death receptor)
DD: (death domain)
DED: (death effector domain)
AQUA: (absolute quantification)
